# Diplochorous Seed Dispersal of Rafflesiaceae by Fruit‐Consuming Squirrels, Rats and Treeshrews, Then Seed‐Burying Dung Beetles

**DOI:** 10.1002/ece3.74467

**Published:** 2026-10-26

**Authors:** Hans Bänziger, Kimio Masumoto, Chainupon Suwannakunpaisan, Stephen Elliott

**Affiliations:** ^1^ Department of Entomology and Plant Pathology, Faculty of Agriculture Chiang Mai University Chiang Mai Thailand; ^2^ Thailand Natural History Museum, National Science Museum Pathum Thani Thailand; ^3^ The Forest Restoration Research Unit, Biology Department, Faculty of Science Chiang Mai University Chiang Mai Thailand; ^4^ Retired

**Keywords:** endozoochory, exozoochory, *Sapria himalayana*, scarabaeid microphagy

## Abstract

Understanding seed‐dispersal of Rafflesiaceae species is essential to conserve this extraordinary family of parasitic plants. We present evidence supporting diplochorous seed dispersal, involving primary endozoochorous dispersal by small mammals, followed by secondary exozoochorous dispersal by dung beetles. Methods employed included in situ live capture of small mammals in traps, placed over developing *Sapria himalayana* fruits, in situ observations of dung beetles handling the small dung pellets of primary dispersers and ex situ experiments with both dispersers. Dung pellet analyses (number, weight, expulsion time and ratio of undamaged to broken seeds) indicated that the most capable primary seed dispersers were the nocturnal rat *Maxomys surifer* (among four species studied) and, during daytime, the squirrel *Dremomys rufigenis* and, to a lesser extent, the treeshrew *Tupaia belangeri* . Dispersal distances were likely to encompass multiple host *Tetrastigma* spp. (lianas). Dung beetles served as secondary dispersers. They removed all faecal pellets of primary dispersers from the soil surface within 24 h, burying them up to 7 cm deep. The beetles also released seeds from the faecal matrix by microphagy, enabling the seeds to come into intimate contact with hosts' fine roots and root hairs, through which infection likely occurs. Forty‐one beetle species (including seven new species) were involved, with *Ochicanthon thai* and *Onthophagus lindaae* being the most abundant. Further research throughout the distribution of Rafflesiaceae species, particularly in the Philippines, regarded as a centre of *Rafflesia* diversity, is likely to add to primary and secondary disperser diversity.

## Graphical abstract

Evidence supporting diplochorous seed dispersal of Rafflesiaceae species is presented. Rodents and treeshrews ingest fruit and act as primary dispersers. Dung beetles subsequently burry and disassemble seed‐laden faecal pellets, potentially placing seeds in close proximity to the roots of host *Tetrastigma lianas*. Implications for conservation of this enigmatic family of parasitic plants are discussed.

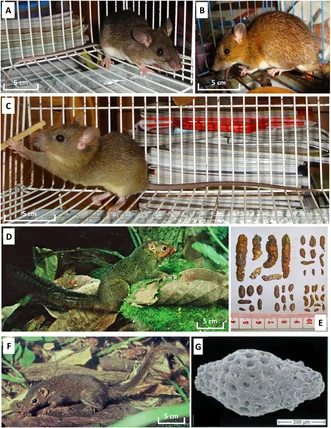

## 1 Introduction

Plant species in the family Rafflesiaceae are renowned for their huge flowers, parasitic habit and extraordinary, but poorly understood, life cycles. All Rafflesiaceae species are holoparasites, being entirely dependent on host plants for energy, nutrients and water. Their hosts are a few liana species in the genus *Tetrastigma* (Vitaceae) (Pelser et al. 2016), in the tropical and sub‐tropical forests of southeast Asia, from the eastern Sub‐Himalayas down to Java and the Philippines. The vegetative parts of these parasitic plants are usually described as mycelium‐like strands that infiltrate host tissues (Kuijt 1969; Meijer 1997) or uniseriate strands of cells with large cell‐nuclei (Brown 1912), but see also Ng's (2025) ‘protocorm’ theory.

The Rafflesiaceae currently consists of three genera: *Rafflesia* (comprising 42 accepted species), *Sapria* and *Rhizanthes*, both comprising four accepted species each (POWO 2025). However, the discovery of new species continues, particularly in the Philippines (Tobias et al. 2024). Thailand is unique in being the only country where all three Rafflesiaceae genera can be found (Figure 1).

**FIGURE 1:**
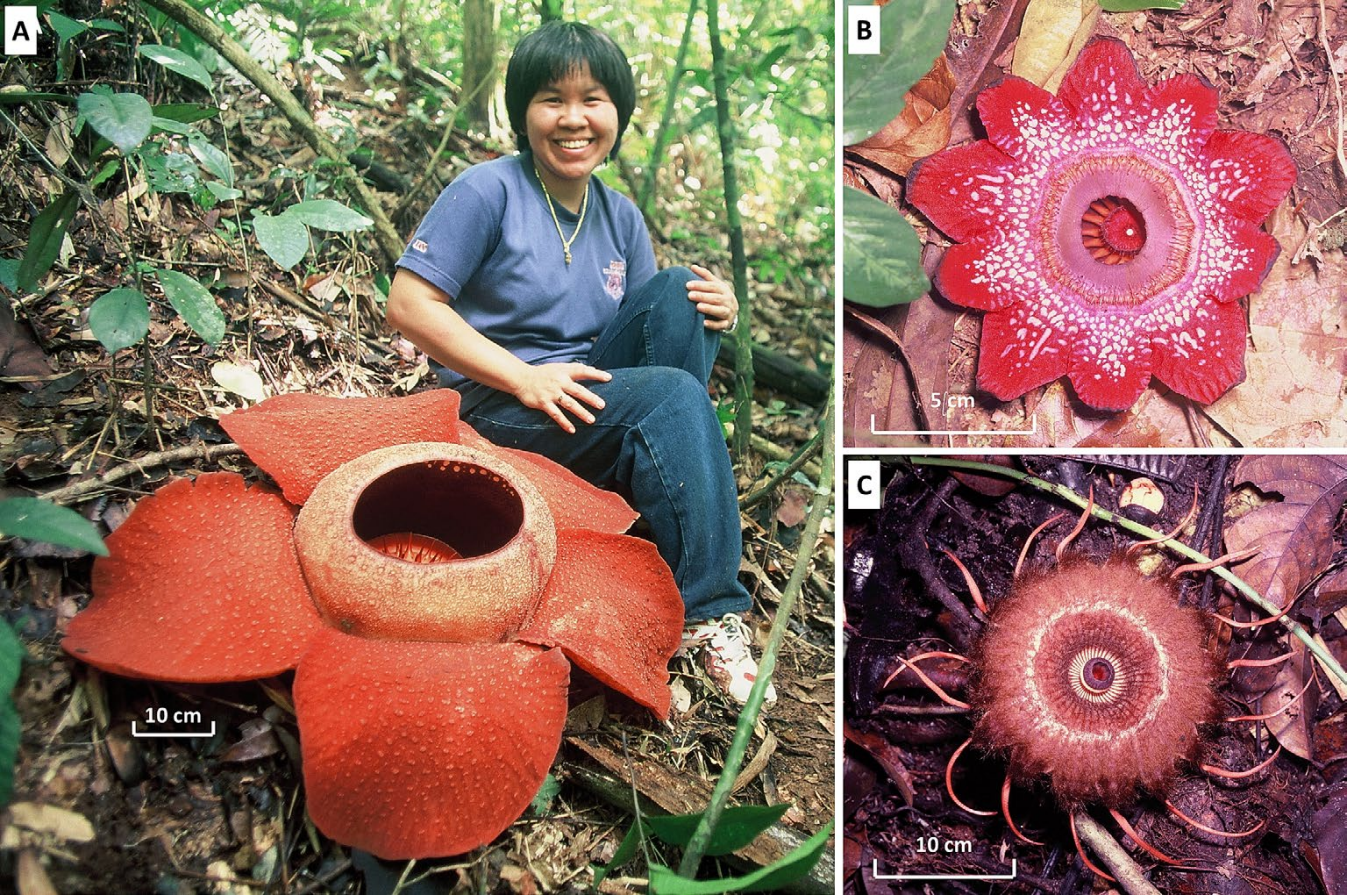
Thailand is the only country where all three of Rafflesiaceae genera are represented: (A) *Rafflesia kerrii* (S Thailand, W Malaysia) (Photo H. Bänziger, in Johnson and Schiestl 2016); (B) *Sapria ram* (endemic to Thailand) (Photo H. Bänziger, in Bänziger and Hansen 1997) and (C) *Rhizanthes infanticida* (S Thailand, W Malaysia, Indonesia (W Sumatra)) (Photo H. Bänziger, in Bänziger 2001).

Although the family has not been comprehensively assessed for the International Union for the Conservation of Nature's (IUCN) Red List (only *Rafflesia magnifica* Madulid, Tandang and Agoo is currently listed as critically endangered (Madulid et al. 2008)), evidence is growing that up to 60% of *Rafflesia* species may be at risk of extinction, since many of their known locations are outside protected areas (Malabrigo et al. 2025). Within Thailand, *Sapria ram* Bänziger & Hansen (Figure 1B) is considered ‘endangered’ nationally (Santisuk et al. 2006).

This paper focusses primarily on seed dispersal of *Sapria himalayana* Griff. in northern Thailand, although we also draw on our experiences of secondary seed dispersal of the other two Rafflesiaceae genera in southern Thailand and northern Borneo (E. Malaysia). Introductory information about the species involved follows.

### 1.1 Distribution and Habitat

*S. himalayana* (Figure 2A) grows in old‐growth, hill‐evergreen forest, mostly at elevations from 1000 to 1450 m asl (Elliott 1992). The species was first described by Griffith (1845) from specimens he collected in 1836 from the Mishmee Hills, Arunachal Pradesh (formerly Upper Assam). It has also been reported elsewhere in India (Syiemiong et al. 2022; Devi et al. 2022; Ralte et al. 2022), SW China (Yunnan) (Li 1975), SE Bhutan (Dorji et al. 2022), Myanmar (Tanaka et al. 2019), Laos (Guo et al. 2023), S Vietnam (Trần et al. 2018) and Thailand (Hosseus 1907; Elliott 1992; Bänziger et al. 2000; Bӓnziger and Pape 2004).

**FIGURE 2:**
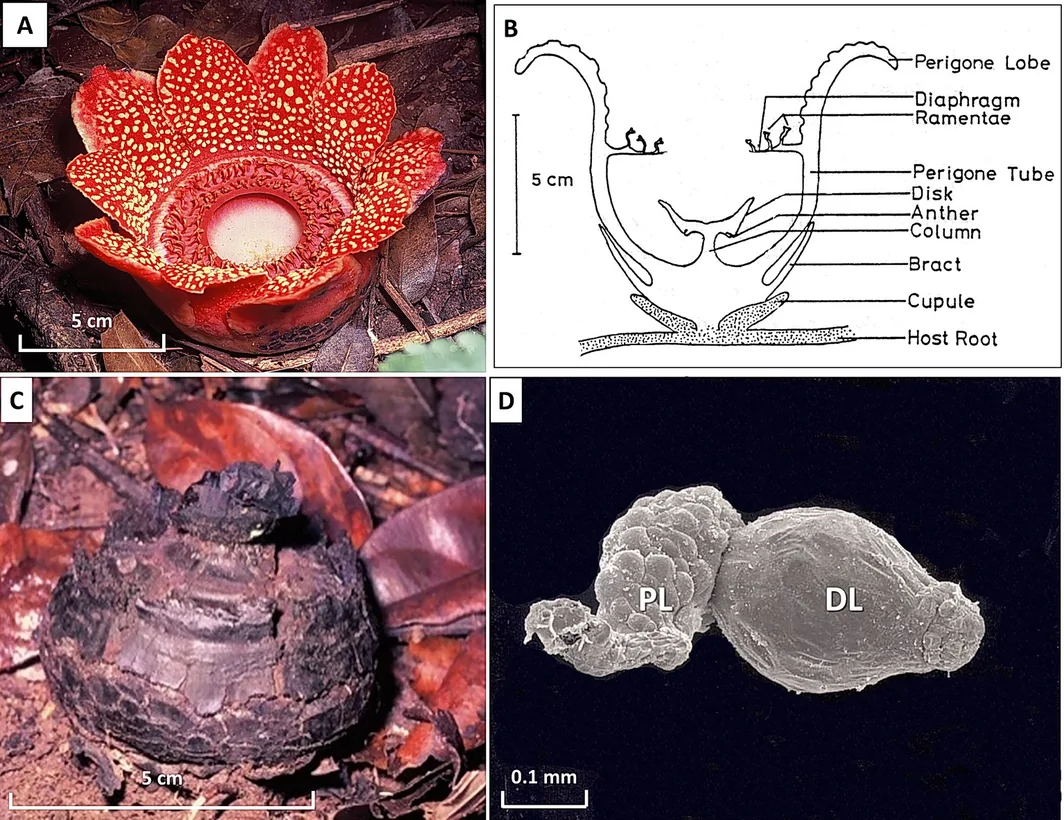
(A) *Sapria himalayana* Griff. open female flower (Photo: H. Bänziger); (B) longitudinal‐section of a male flower, showing morphological terms for constituent parts (from Elliott 1992); (C) a mature *S. himalayana* fruit (156 days after pollination) (Photo: H. Bänziger) and (D) an electron micrograph image of a *S. himalayana* seed (A. Blarer in Bӓnziger 2004), DL, distal lobe containing embryo; PL, proximal lobe (various connective tissues).

In Thailand, *Ra. kerrii* (Figure 1A) is present southward from 200 km north of the Kra Isthmus, down to northernmost W Malaysia at elevations of 475–840 m asl also in lowland evergreen rain forest. *Rhizanthes lowii* (Becc.) Harms is endemic to Borneo (Bӓnziger et al. 2007).

Habitat‐ecological data (soil, climate; bud development, mortality, host lianas) for *S. himalayana* were provided by Elliott (1992) and Syiemiong et al. (2022) and for *Ra. kerrii*, by Meijer and Elliott (1990) and Bӓnziger (1991).

### 1.2 Reproductive Ecology

Interest in Rafflesiaceae species has grown markedly among researchers over the past two decades, particularly their reproductive ecology, to conserve species and display them in botanical gardens.

Rafflesiaceae pollinators (Figure 3) and pollination mechanism are summarised in Appendix 1. Pollen is presented as a viscous suspension, which adheres to the back of pollinating flies, but only when fluid under conditions of high humidity. Within *S. himalayana* flowers, during N Thailand's hot‐dry season in (March to April), high humidity is achieved by rapid transpiration of water vapour, which also cools the perigone‐tube interior (Figure 2B) (Bӓnziger et al. 2022). In contrast, during the rainy season, *S. himalayana* flowers are slightly thermogenic, as for other Rafflesiaceae in ever‐wet regions (Patiño et al. 2000, 2002).

**FIGURE 3:**
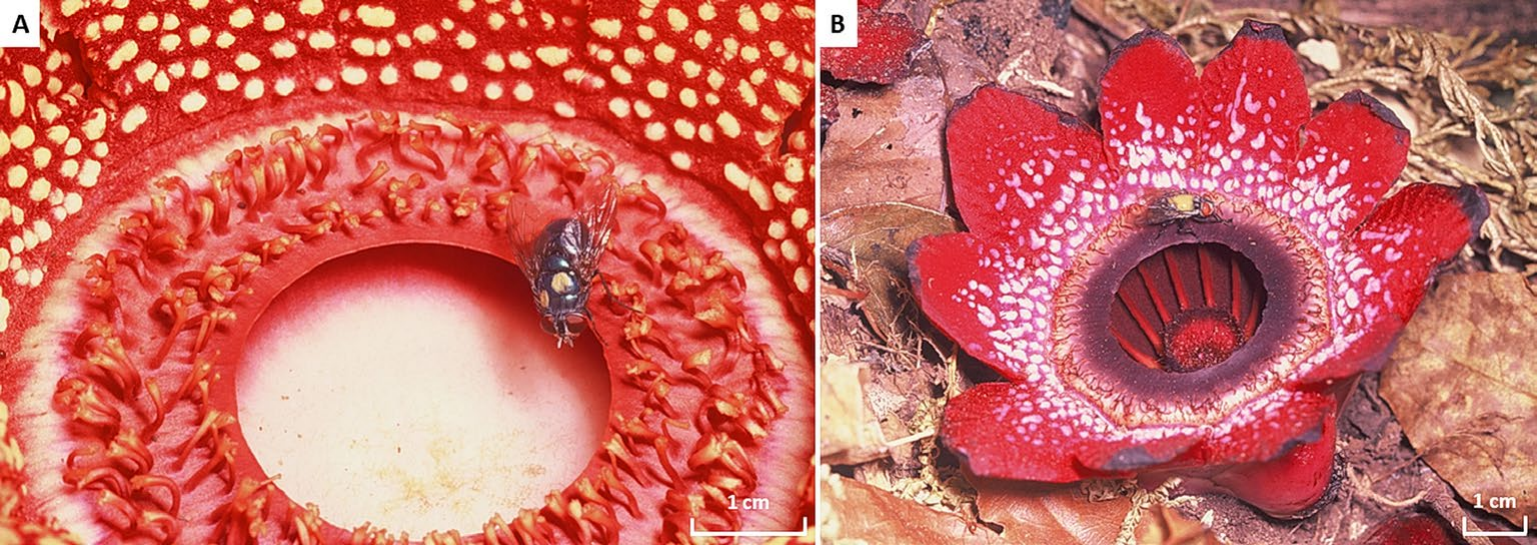
(A) A female *Lucilia porphyrina* with two dry pollen smears on the thorax (from previous visit to a male flower) ready to pollinate a female flower of *S. himalayana* and (B) A female *Sarcophaga saprianovae* emerged from a male flower of *S. ram*, having acquired a yellow pollen smear by circumambulating the underside of the disc (Photos: H. Bӓnziger).

Development of fruits and seeds (Figure 2C,D) has been described for *S. himalayana* (Bӓnziger 2004). A comparison of fruit and seed morphological traits of the focal Rafflesiaceae species in this paper is presented in Appendix 2.

A wide range of frugivorous animals have been proposed as seed‐dispersers of Rafflesiaceae species. Justesen (1922) first speculated that termites, wild pigs, pangolins and mice might disperse *Rafflesia* spp. seeds; Kuijt (1969) later added elephants. More recently, Pelser et al. (2013, 2018) proposed that ants could act as primary seed dispersers, based on observations of *Technomyrmex* and *Pheidologeton* species moving seeds in a decaying fruit of *Ra. philippensis* Blanco and *Ra. speciosa* Barcelona and Fermando in the Philippines. Earlier, Meijer (1958) proposed squirrels and treeshrews as exozoochorous dispersers, noting that seeds, carried externally on snouts and claws, might be introduced into host lianas through incidental wounding.

Indirect evidence, supporting endozoochory, was provided by Elliott (1992), who reported rodent tooth marks on 18% of *S. himalayana* fruit remains. Fruit contents had been almost completely removed, leaving only the base of the fruits still attached to the host. Bӓnziger et al. (2007) also reported rodent tooth marks on *Rh. lowii* fruits. Direct evidence came from Emmons et al. (1991), who observed *Tupaia tana* and *Callosciurus notatus* consuming fruits of *Ra. keithii* Meijer in situ. Furthermore, Bӓnziger (2004) demonstrated, under experimental conditions, that *Rattus norvegicus* and *Mus musculus* consume *S. himalayana* fruits, with 85%–90% of seeds passing intact in faecal pellets. Thus, endozoochory, as the primary seed‐dispersal mechanism of Rafflesiaceae species, emerged a plausible hypothesis for further research.

In this paper, we also explore the likelihood that dung beetles act as secondary dispersers of seeds, first deposited in small‐mammal faecal pellets. The supposition that dung beetles act as secondary dispersers arose out of serendipitous observations (by HB) that, when rodent pellets were exposed in situ (to study their break‐down by rain and presumed fly feeding), dung beetles removed and buried the pellets. To our knowledge, diplochory has never been proposed for seed dispersal of Rafflesiaceae; nor have dung beetles previously been implicated as secondary dispersers. However, several studies have shown that dung beetles remove the droppings of medium‐ to large‐sized mammals. These include large rodents such as the agouti (4–9 kg body mass) and the north American prairie dog ( *Cynomys ludovicianus* (ground squirrels of 0.5–1.5 kg)), the droppings of which are removed by *Canthon praticola* (Gordon and Cartwright 1974). However, we found only one reference to scarabaeids removing dung of small mammals (such as rodents and scandents weighing less than 0.3 kg), namely that of a rat by *Panelus* sp. (Paulian 1945).

Here, we focus on a guild of dung beetles that roll intact, small, cylindrical faecal pellets (from small mammals) over short distances. We refer to this guild as ‘non‐balling rollers’ (rather than the ‘non‐rolling rollers’ of Halffter and Halffter 1989), to distinguish them from typical rollers. The latter are often larger beetle species that excise portions of larger, softer dung pats, which they shape into balls, before rolling them to a new location.

The speculative and indirect nature of current evidence of *Rafflesiaceae* spp. seed‐dispersal mechanisms constitutes a critical knowledge gap that hinders the conservation of these threatened parasitic plants, both in situ and in botanical gardens. Therefore, the study presented here aimed to determine, for the first time, the effectiveness of rodents, treeshrews and dung beetles as dispersers of Rafflesiaceae spp. seeds by generating direct evidence from multiple experiments and structured field observations. We evaluated the relative dispersal efficiency of small mammal species according to seed displacement potential, terrestrial activity, seed‐expulsion duration, faecal output and attractiveness of faeces to dung beetles, and investigated the subsequent role of dung beetles in secondary seed dispersal toward host roots as a prerequisite for host infection.

## 2 Study Sites

In northern Thailand, our study sites were located in primary hill evergreen forest within Doi Suthep‐Pui National Park, Chiang Mai Province. We worked on 13 separate ‘clusters’ of *S. himalayana* between 1200 and 1350 m elevation—a cluster being defined as a group of buds, flowers and fruits, parasitizing a single host plant. Host plants were one *Tetrastigma obovatum* (M.A. Lawson) Gagnepain and 12 *T. planicaule* (Hook. f.) Gagnepain (= *T. laoticum* Gagnepain according to Trias‐Blasi et al. 2020) (Vitaceae).

Observations of secondary seed dispersal by dung beetles were also made in tropical evergreen forest in the Thailand Provinces of Ranong (100–200 m asl, two clusters) and Chumpon (200–300 m asl) and at two evergreen rainforest sites (750 and 900 m asl) in northern Borneo (Sabah, E. Malaysia). According to Dr. A. Lamb (in Bӓnziger et al. 2007), the latter areas were private land, hence no government permission was required to collect dung beetles there.

## 3 Materials and Methods

### 3.1 Enhancing Fruit Set

The flowering season of *S. himalayana* is October to April. To obtain sufficient fruits for in situ frugivore trapping and ex situ feeding experiments, *S. himalayana* flowers were manually pollinated using Bӓnziger's (2004) methods. This increased the fruit set rate from a natural level of 8%–12% to 78%, resulting in a net positive effect on the plant's reproductive cycle locally. Over three flowering seasons (January to March/April 2005, 2006, 2007), 179 female flowers were marked with sticks and manually pollinated to produce the projected requirement of 40 fruits (well over the replacement rate) as in situ live bait for trapping frugivores. Ten more were used for feeding experiments *ex situ*. Fruit development took 5–6 ½ months, from pollination to the natural splitting open of the fruit when it is considered overripe. Ripe fruits were mostly found from the end of May until mid‐August (Bӓnziger 2004 and HB pers. obs.).

### 3.2 Mammal Trapping

Live‐trapping of frugivores was by Sherman‐like traps, purchased in a local market, measuring 20 × 20 × 38 cm. Each trap was modified so that it could be secured over a live *S. himalayana* fruit in situ. The rear end of each trap, for a width of 6 cm, was cut from the remaining 32‐cm entrance side so that the two trap sections could be fitted together over a developing fruit. To accommodate the *S. himalayana* fruit, attached to its host liana root within the trap, a 4 cm diameter disk was cut out from the trap base, where the two sections were joined. The trap trigger was a bamboo cross 15 cm wide which, hanging from the upper side of the trap halfway inside, obstructed any animal's approach to the fruit. If an animal displaced the cross, even slightly, the trap door would spring shut, capturing the animal inside. Once the two trap parts were correctly assembled over a fruit, they were firmly joined using wire tethers on the sides and top of the trap. The trap was firmly fixed to the ground by two 30‐cm‐long rods, recurved at the top, to hook onto the trap base, pierced into the soil (Figure 4).

**FIGURE 4:**
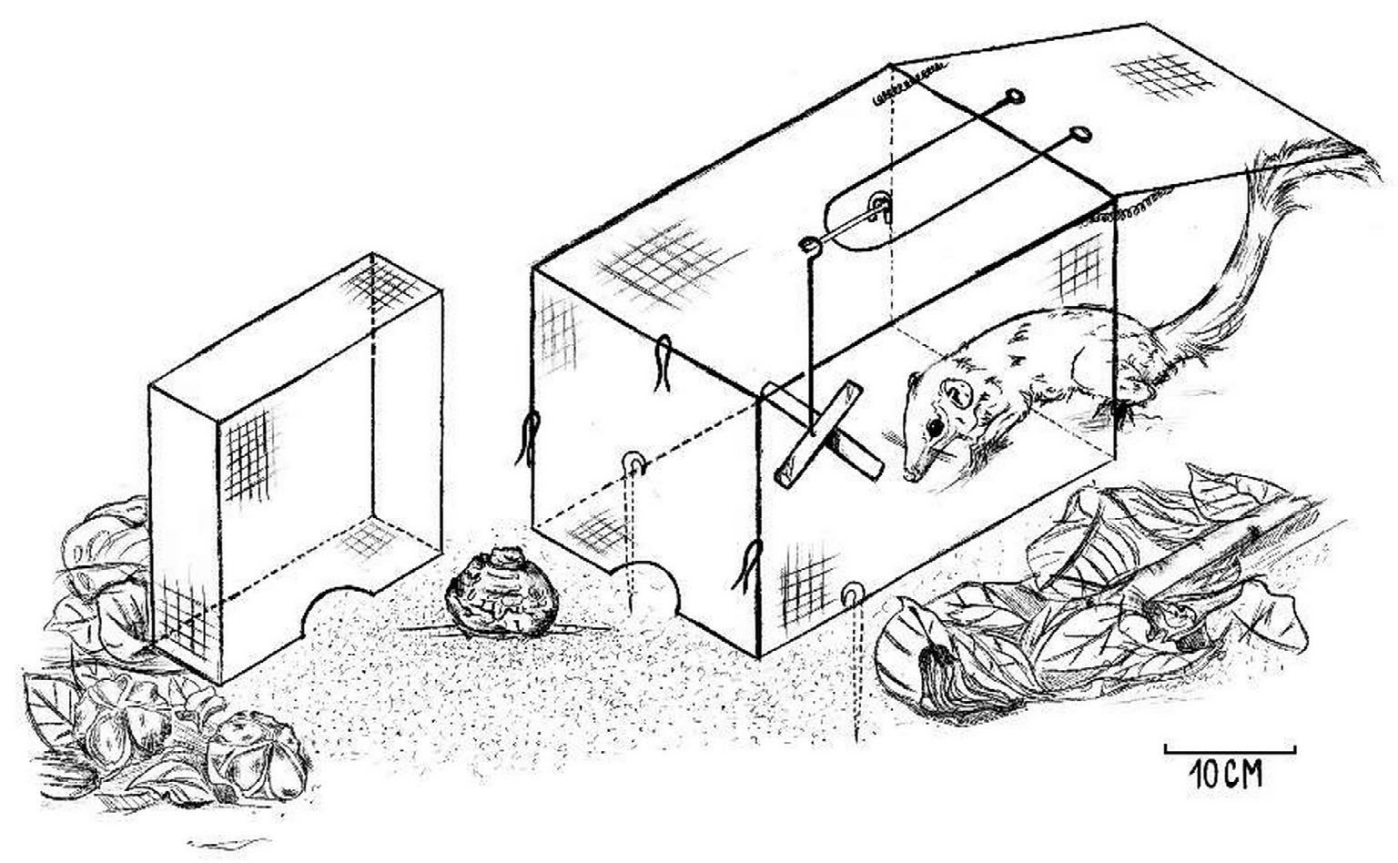
Setting up a mammal trap over a live fruit of *Sapria himalayana* in situ. The end section left was joined to the main section (right) using the three wire ties illustrated (drawn by Atcharawan Saeaiew).

Traps remained in position for several weeks, as the fruit matured, since rats and treeshrews are known to eat both ripe and non‐ripe fruits. Traps were disassembled and removed when fruits began to break up and decay (Bӓnziger 2004). Since the flowers had been manually pollinated, the age of each fruit was known exactly. Up to four traps were active simultaneously, placed far enough apart as to be out of view from each other (50–100 m apart, depending on vegetation density, but some > 1 km apart).

Traps were inspected daily or on alternate days. Therefore, each animal remained in the trap for less than 36 h between capture and release. During this period, each animal consumed *S. himalayana* fruit, defaecating the seeds in pellets, which fell through the wide mesh of the trap onto the soil beneath, thus further minimising the effects of trapping on the plant's reproductive cycle.

Captured animals were released, except for a single specimen per species, which was temporarily taken to the lab for studies of ingestion, digestion and defaecation. Furthermore, in the case of rats, skull specimens were prepared for taxonomic identification. Animals were transported to the lab, either still contained in traps, or they were extracted from the traps into black cloth bags in the field. The cupula of the *S. himalayana* fruit (connecting it to the host root) was cut below the trap and adjacent to the trap floor but above the root. The trap was then placed in a black cloth bag and the entire trap transported to the lab. Alternatively, the bag was placed over the trap entrance, which was then opened, so the animal could exit and retire into the dark recess of the sack, which was firmly tied closed. The trap could then be placed over a new *S. himalayana* fruit and reset. Murids were identified by CS.

### 3.3 Captive Feeding Experiments

In the lab, the animals that were used in *ex situ* feeding experiments were weighed and housed in regular cages measuring 45 × 70 × 110 cm. Tree branches were placed inside the cages to allow the animals to climb, as well as a small shelter to provide security when sleeping.

Multiple captive feeding experiments with an individual of each species were performed to determine how quickly *S. himalayana* seeds pass through their digestive systems, in what quantities and how many emerged without having been crushed during mastication. The captive mammals were first acclimatised to a diet consisting of rat chow, supplemented with sunflower seeds, bananas and plenty of mealworms for periods of 5–20 days (depending on species). Water was provided ad libitum. This procedure flushed out any *Sapria* seeds, which may have been in their gastro‐intestinal tract.

Feeding experiments were then initiated by providing an irregular, roughly cubic piece of *Sapria* fruit, measuring 2–3 cm on each side to each animal. Each piece of fruit was estimated to contain 4000–5000 seeds. The seeds were tiny (0.6–0.65 mm long by 0.29–0.33 mm wide (Figure 2D)) and very irregularly distributed within the fruit. The fruit was usually consumed over 5–15 min (the half‐way time was taken as the ingestion time). Subsequently, the usual food and water were given as mentioned.

Faecal pellets were collected and kept at about 10°C, when not immediately analysed for seeds. Collection frequency varied according the expulsion rate (see online data). The pellets were first macerated with water before examination under a stereomicroscope (magnification 40×) for counting and damage assessment. Damaged seeds were those showing obvious fissures or breakage. Seed fragments could not be counted. For determination of pellet dry weight, all pellets, expelled each day per animal, were first placed in a sealed container with silica gel for a week, to ensure complete dryness. The weight of the daily sample was then divided by the number of pellets, to derive a mean value.

Besides those mammals, captured whilst consuming *S. himalayana* fruits, two others, which were not trapped with *Sapria*‐fruit bait (despite being commonly observed in *Sapria* habitat) were also captured with non‐modified Sherman traps, baited with banana: the Pallas's squirrel ( *Callosciurus erythraeus* ) and the Indochinese ground squirrel ( *Menetes berdmorei* ). The purpose of bringing them into captivity was to determine if they would consume *Sapria* fruits, albeit under *ex situ* conditions. *C. erythraeus* was trapped in a garden at the foot of Doi Suthep outside the National Park. This animal was also used, to obtain sufficient faecal pellets to be used as bait for field research on dung beetles.

### 3.4 Field Observations and Trapping of Dung Beetles

Data on the secondary seed dispersers—dung beetles—were obtained by two methods. The most important one was by direct observation, to determine how beetles processed, transported and buried the pellets. The other was by pitfall trapping. The data‐collection effort, expended on both techniques, is summarised in Table 1.

**TABLE 1:** Observation periods and trapping effort of dung beetles (mm/yy).

Region and focal species	Study sites	Direct observation effort	Trapping effort (Pitfall traps)
Northern Thailand *S. himalayana*	Chiang Mai Province	96 h (40 sessions)^a^ 08/05–01/06, 05–11/06, 08/07	6 days 11/06, 12/06 (24 h each)
Southern Thailand *Ra. kerrii* & *S. ram*	Ranong & Chumpon Provinces	29 h (6 sessions) 09/05, 02–03/06, 02/07, 08/07	7 days 02/07, 08/07 (24 h each)
Northern Borneo (Sabah) *Rh. lowii*	Ulu Moyog, Penempang Distr.	20 h (9 sessions) 01/06, 02/06	None

^a^ Includes 2 h (4 sessions) at dusk and 6 h (3 sessions) at night; all other sessions were diurnal.

For the first method, one to several faecal pellets from the primary seed dispersers were placed on the soil surface or on a leaf and the behaviour of any beetles arriving in the vicinity of the bait was recorded. The beetles were subsequently collected, anaesthetized by ethyl acetate, set (pinned or glued on paper points) for identification under magnification, and kept as vouchers. Over 55 sessions, 145 h of observations were recorded, mostly during the day, but including 8 h at dusk and night (Table 1).

For the second method, we used plastic cups, 9 cm in diameter, 5 cm high, with a bait receptacle in the centre—a plastic disc 2.5 cm in diameter with a rim 0.5 cm high, glued to the top of a 5‐cm‐high plastic column and two rain water release windows 0.5 × 1 cm, covered by bronze mesh, set 0.5 cm below cup ridge. The cup was buried, leaving around 0.5–1 cm protruding above the soil surface and half‐filled with water. The bait—one to several faecal pellets—was placed on the receptacle. Trapped dung beetles were either found drowned or, less commonly, swimming on the water surface. The inner surface of the cup was too smooth to enable them to climb out. After about 24 h, trapped beetles were collected as above. Identification was performed mostly by KM (who was not involved in the field studies).

Collected Thai specimens, including the holotypes, are deposited at HB's Entomology Department Collection (CMU). The holotype and some paratypes of the Malaysian species are deposited at the National Museum of Natural Science, Tokyo, Japan; the other paratypes are split between CMU and the Sandakan Herbarium Sabah, Malaysia where HB's previous plant specimens are also housed.

### 3.5 Lab Experiment—Faecal Pellet Processing by Dung Beetles

Ex situ experiments assessed the degree and speed with which the seeds of *S. himalayana* were disaggregated from the faecal pellets by scarabaeid microphagy. Circular plastic boxes (diameter 8 cm, height 4 cm) were prepared, each with an aeration opening (2 × 2 cm), covered by bronze mesh, in the cap. Five trials were performed, each with 1 box containing one live *Onthophagus avocetta* (Figure 7D) (four with males and one with a female) and one faecal pellet. This large‐bodied species was selected to determine if seed ingestion was possible. All beetles were collected from the study sites. Fresh faecal pellets were harvested from a caged *M. surifer* (Figure 5A) shortly before the experiments were begun. Each pellet contained an unknown number of seeds from a piece of *S. himalayana* fruit, provided to the rat 24 h previously, plus chow and mealworms (as described above). Experiments commenced at 10 pm. The boxes were placed in the dark for 24 h, after which they were opened and the contents examined.

**FIGURE 5:**
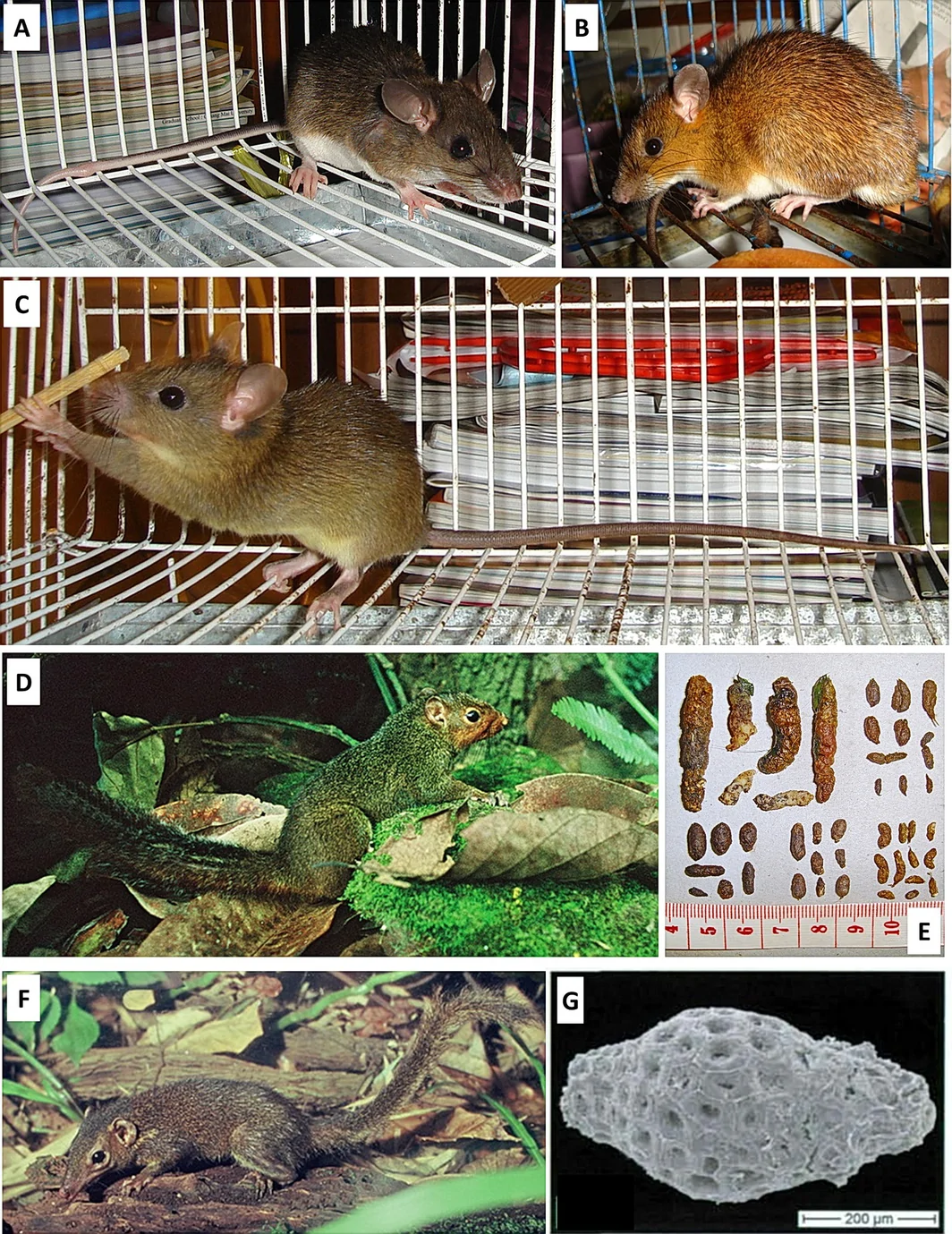
(A) *Maxomys surifer*; (B) *Niviventer fulvescens*; (C) *Chiromyscus langbianis*; (D) *Dremomys rufigenis*; (E) The disproportionate size of the faecal pellets of *Tupaia belangeri* (top left) compared with those of the four rodent species, *D. rufigenis* (top right), *M. surifer* (bottom left), *C. langbianis* (bottom centre), and *N. fulvescens* (bottom right); (F) *T. belangeri* (Photos: H. Bänziger) and (G) An electron micrograph image of an intact *S. himalayana* seed, extracted from a faecal pellet, showing exposed pitted exotegmen and sparse remnants of the distal lobe (to the right) (A. Blarer in Bӓnziger 2004).

## 4 Results

### 4.1 Trapped Mammalian Frugivores (Primary Dispersers)

Six small mammal species were captured in traps secured over live *S. himalayana* fruits in situ, including four rat species, one ground squirrel and one treeshrew. All species consumed *S. himalayana* fruits. Details of the animals captured are listed in Table 2. Observations from cages in the lab revealed that the animals ate the fruits by stabbing their lower incisors (four in *Tupaia*, two in rodents) into the fruit, leaving a mark of four or two channels dug into the flesh.

**TABLE 2:** Mammals captured in traps over *S. himalayana* fruit in situ.

Order	Family	Species	Common name	No. animals trapped	Date
Scandentia	Tupaiidae	*Tupaia belangeri*	Northern Treeshrew	4	28/05, 15, 21, 23/06/06
Rodentia	Sciuridae	*Dremomys rufigenis*	Red‐cheeked Ground Squirrel	4	06/07/05, 21/06/06, 18, 20/08/07
Rodentia	Muridae	*Maxomys surifer*	Indomalayan Spiny Rat	1	04/07/06
Rodentia	Muridae	*Chiromyscus langbianis*	Langbian Tree Rat	1	12/07/06
Rodentia	Muridae	*Niviventer fulvescens*	Chestnut White‐bellied Rat	1	20/07/06
Rodentia	Muridae	*Rattus andamanensis*	Indochinese Forest Rat	1	29/06/07

### 4.2 Seed Passage Through the Digestive Tracts of Small Mammals

The rodent species produced firm, faecal pellets of similar size and water content, whereas *T. belangeri* (Figure 5F) produced much larger soft pellets (Figure 5E), with high water content (Figure 6).

**FIGURE 6:**
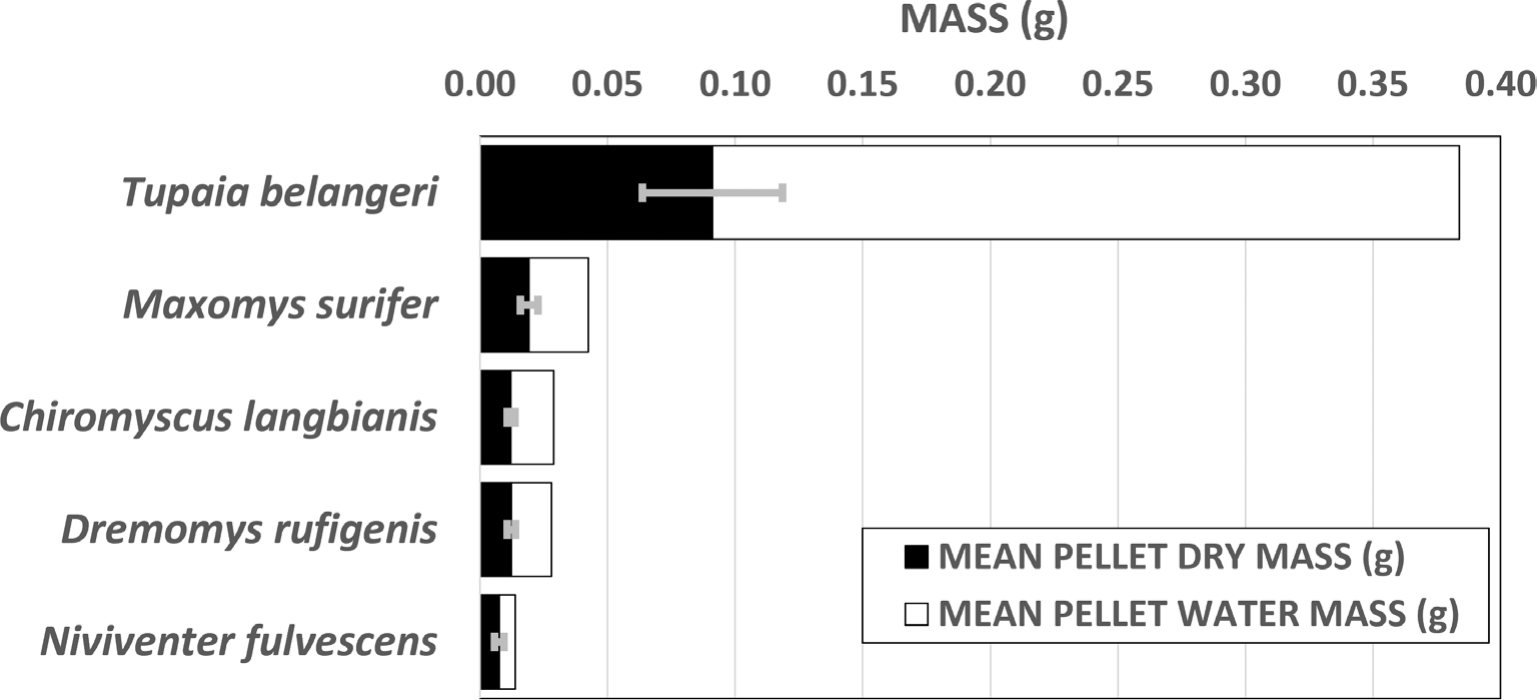
Mean dry mass (error bars are standard deviations) and water content of individual faecal pellets of small mammals in captivity after consuming fruit pulp of *S. himalayana* .

Seeds began to appear in the faeces of the mammals 1–15 h after feeding. Seed expulsion rates (seeds/h) peaked 1–26 h after ingestion with the last seeds being expelled up to 94 h after the first (*M. surifer*, Figure 5A). The vast majority of seeds from faecal pellets, examined microscopically, appeared undamaged, with broken seeds amounting to only 0%–13% of the total (Table 3).

**TABLE 3:** Faecal pellets and seeds expelled from small mammal species (all adults), fed ripe fruit pulp of *S. himalayana* in captivity.

Species	Treeshrew	Squirrel	Rats		
	*T. belangeri*	*D. rufigenis*	*M. surifer*	*C. langbianis*	*N. fulvescens*
Body mass (g)	153 ♂	198 ♀	106 ♀	97 ♀	116 ♂
No. experiments^a^	3	4	2	3	4
Faecal pellets					
Mean number expelled per 24 h	16.3	121.1	20.3	39.4	69.4
SD	6.4	22.1	4.7	7.8	11.2
Mean dry mass of pellets expelled per 24 h (g)	1.377	1.472	0.380	0.484	0.540
SD	0.403	0.179	0.057	0.124	0.190
Seed expulsion					
Time to first seed (h)	< 1.2–2.3	1.4–5.3	6.3–15.1	1.6–2.0	1.7–3.0
Time (h) to maximum seed‐expulsion rate^b^ (seeds/h)	1.5–3.8	2.4–8.0	26.2–26.3	3.1–9.6	2.5–9.76
Time to last seed (h)	6.5–21.0	8.7–> 34	100.2–100.5	9.7–32.0	14.0–75.2
Mean % of expelled seeds non‐damaged not broken	99.9	86.9	99.9	99.97	98.8
SD	0.22	8.16	0.12	0.06	0.72

Abbreviation: SD, standard deviation.

^a^ Multiple experiments on single animals of each species.

^b^ Peak seed expulsion rates (seeds/h) read from graphs presented in the online data.

Gut passage of seeds through the treeshrew ( *T. belangeri* ) was the most rapid, with almost no damage to the seeds (99.9% emerging intact).

The squirrel ( *D. rufigenis* , Figure 5D) stood out as expelling the largest number and mass of faecal pellets. Passage of seeds through the digestive tract was more rapid than that of the rat species, but slightly slower than that of the treeshrew. The percentage of damaged seed was highest for the squirrel, compared with the other species. However, with only 13% of passed seeds recorded as damaged, the figure was still very low.

Of the three rat species, *N. fulvescens* (Figure 5B), passed the greatest mass and number of faecal pellets, also relatively rapidly. *M. surifer* (Figure 5A) exhibited the longest duration of seed expulsion from 6 to 100 h following ingestion. This would result in dispersal over long distances, albeit at lower densities (compared with the other rats), due to the relatively lower numbers and mass of faecal pellets produced (per 24 h).

Rafflesiaceae seeds are bilobed. The larger, distal lobe contains the embryo, whereas the smaller, proximal lobe consists of residual tissues that originally connected the developing seed to the ovary wall—namely the outer integument (testa), chalaza, raphe and funicle (Figure 2D) (Bouman and Meijer 1994). Microscopic examination of the expelled seeds (SEM ×100–200) revealed removal of the proximal lobe and outer seed coverings during gut passage (comparing Figures 2D and 5G). However, the vast majority of seeds survived mastication and digestion intact and hard (tested by squeezing with forceps) (Table 3). Consequently, passage through the digestive tract of the primary seed dispersers did not prevent the expulsion of large numbers of intact seeds.

The maximum seed load per faecal pellet varied widely by species, ultimately constrained by pellet size. The large, moist pellets of *T. belangeri* (Figures 5E and 6) contained up to 1500 seeds/pellet during peak seed‐expulsion periods. In contrast, the smaller, firmer pellets of the rats—*C. langbianis*, *M. surifer* and *N. fulvescens* (Figure 5C, A and B respectively)*—*carried substantially lower peak loads: 9–95, 86–135 and 24–163 seeds/pellet, respectively. The prolifically produced squirrel faecal pellets carried peak loads of 75–186 seeds/pellet. These findings demonstrate the capability of small mammals to disperse large numbers of intact seeds over periods of up to 100 h, although the quality of dispersal would vary with species. Dispersal by *T. belangeri*, for example, is likely to result in a more clumped dispersion pattern, whereas that of the rodents is likely to be more diffuse.

### 4.3 Evidence for Secondary Seed Dispersal by Dung Beetles

The beetles were highly efficient at locating and removing the faecal pellets from the soil surface in situ. Over most observational periods, all pellets were removed within 24 h. A total of 41 dung beetle species (5 known to genus level only) were rapidly attracted to the faecal pellets of the primary seed dispersers in situ, moving and burying most of them within a few hours. Beetle species names and numbers of observations are listed in Appendix 3.

In northern Thailand, at *S. himalayana* sites, a total of 21 dung beetle species (408 individuals) were collected. Of these, 19 species (220 individuals) were observed on the soil surface, burying faecal pellets, and 16 (188 individuals) were collected from traps. In southern Thailand, at the *Ra. kerrii* and *S. ram* sites, 20 species (114 individuals) were collected, of which 10 (47 individuals) were observed burying rodent pellets on the soil surface and 15 (67 individuals) were collected from traps.

Only three species were present at both the northern and southern Thailand sites (*Oc. thai* (Figure 7B), *On. baenzigeri* and *On. lindaae* (Figure 7C)), reflecting taxonomically distinct but functionally similar beetle communities in both locations.

**FIGURE 7:**
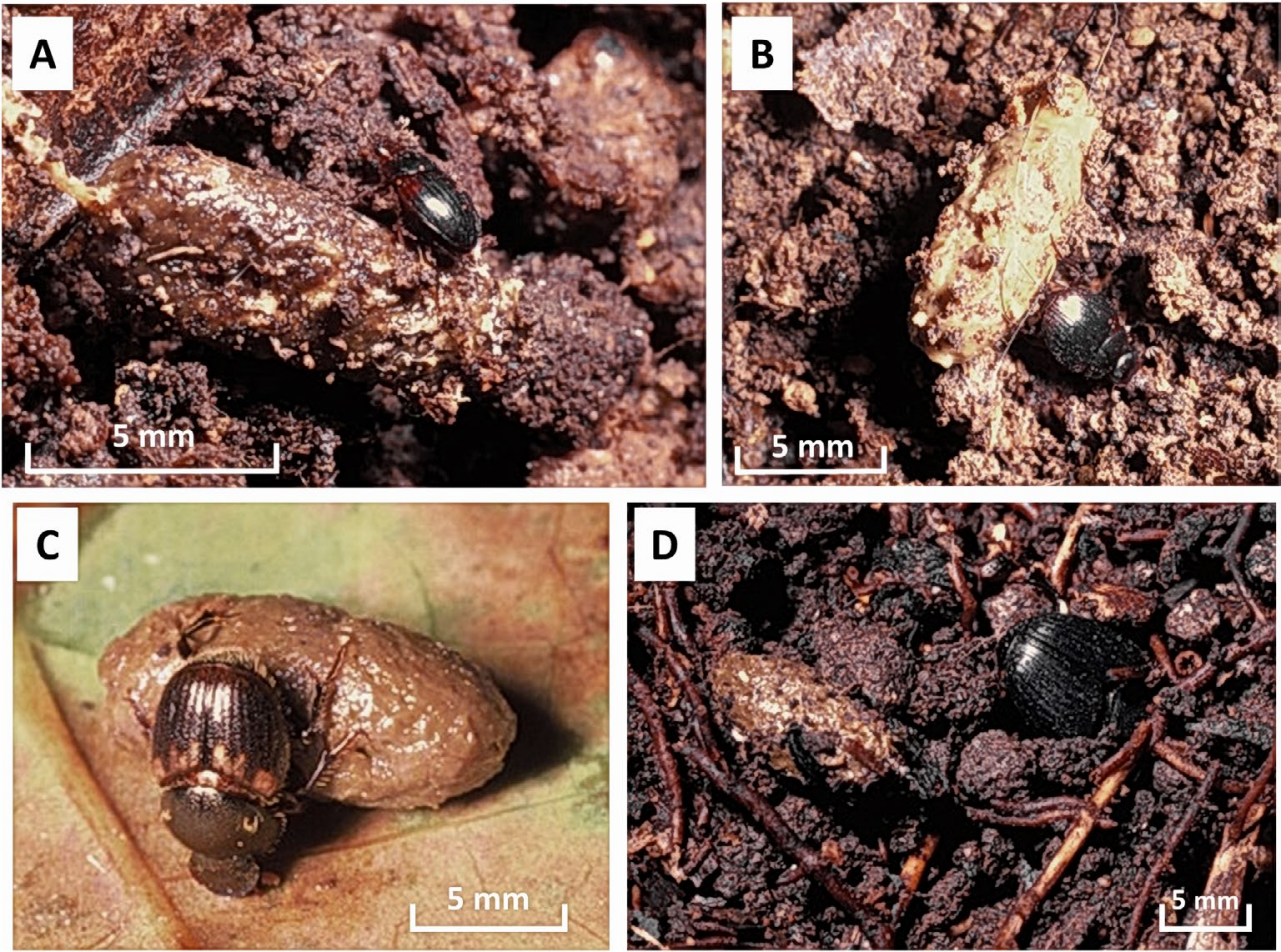
(A) *Panelus tonkinensis* rolling a pellet of *Callosciurus erythraeus* . (B) *Ochicanthon thai* rolling a pellet of *Callosciurus erythraeus* . (C) *Onthophagus lindaae* rolling a pellet of *Maxomys surifer* . (D) After rolling a pellet of *Maxomys surifer* , *Onthophagus avocetta* (only distal half visible), digging itself down preparing to bury it. (Photos: H. Bӓnziger).

In N Borneo at *Rh. lowii* sites, only three species (11 individuals) were observed burying pellets over a very limited time period without traps (20 h, Table 1). None of them were the same as those collected at either of the Thailand sites.

In northern Thailand, 11 species were present when *S. himalayana* was ripe. Most were rare (< 10 observations). Only *Oc. thai* (Figure 7B), *On. avocetta* (Figure 7D), *On. doisuthepensis*, *On. lannamiibun*, *On. lindaae* (Figure 7C), *Panelus tonkinensis* (Figure 7A) and *Parascatonomus maesaensis* were observed in substantial numbers (84, 23, 23, 25, 52, 18, 18, respectively). In southern Thailand, due to the much shorter study period, only *On. lindaae*, *On. ranongensis* and *Sisyphus thoracicus* were commonly observed (with 13, 39, 20 specimens, respectively).

Beetles typically arrived at pellets by flight, either circling briefly or landing 3–20 cm away from a pellet. Most beetles arrived 30–60 min after pellet placement, although many arrived within 2–10 min. Shortest arrival time was 2 min; longest was 7 h 40 min.

Within a few seconds after landing, the beetles crawled toward the pellets and exhibited various behaviours.

Most commonly, all 29 species, collected from the soil surface, were observed rolling whole, intact rodent faecal pellets backwards using, their hind legs, 5–20 cm, over a period of up to 10 min—archetypal non‐balling roller behaviour. The exceptional maximum distance was 100 cm, over 2 h, by an *Oc. thai*, with several interruptions, when it would ‘freeze’, feigning death, for 5–15 min. This occurred when the beetle was disturbed by the arrival of a much bigger beetle (e.g., *On. avocetta*) or by unintended movement of the observer.

Two specimens only, one *On. suthepensis* and one *Par. maesaensis* did not move pellets at all but buried them immediately.

Some *On. avocetta* (Figure 7D) individuals adopted a different approach. They landed approximately 20 cm from a pellet and tunnelled horizontally toward it, just beneath the soil surface. This trajectory was traceable by slight displacement of the soil surface, along with the beetle emerging intermittently, to check and correct its direction. Upon reaching the pellet, the beetle immediately buried it (without rolling).

The larger faeces of *T. belangeri* (Figure 5E) were handled differently. One *On. lindaae* and one *Oc. thai* were observed working for about half an hour, forming excised pieces of pellets into balls before rolling them—characteristic behaviour of typical rollers.

When ready to bury its pellet, each beetle would dig itself into the soil, disappearing completely until soil particles around and below the pellet could be seen moving. From beneath, the beetle would first slowly turn the pellet, one end down, while making it sink into the soil—a process completed within a few minutes. Most species buried faecal pellets at depths of 2–3 cm; however, *On. taayai* and *On. lindaae* reached 4 cm, while *On. orientalis* and *On. ranongensis* achieved depths of 6–7 cm. These measurements, taken 5–10 min after burial, likely represent minimum depths, as further downward movement may occur over time. While field constraints necessitated diurnal collection for most taxa, only *On. taayai* and *On. grandivigilans* appeared strictly nocturnal, whereas *Oc. thai, On. avocetta, On. lindaae* and *S. maniti* were active during both day and night. This is likely to be the case for many other species. However, nocturnal observations were too few for confirmation.

No pair bonding between the two sexes was observed during rolling or burying. Slightly more females than males were collected. Oviposition, which occurs in the nest burrow, was not studied. However, dung balls are not solely for their young. On emergence from pupae, the beetles must first feed themselves during a maturation period lasting several weeks to months, before starting reproduction (Halffter and Matthews 1966).

Post‐burial processing of the pellets by the beetles could not be observed in nature. However, in the lab, when *On. avocetta* beetles were placed with seed‐laden *M. surifer* pellets they completely disassembled them, releasing intact *S. himalayana* seeds from the faecal matrix without ingesting them. The numbers of seeds recovered from each of the five experiments were 15, 11, 26, 28 and 30, distributed singly or in groups. Small, slurry‐like faeces of the beetles contained some extremely small, chitin epidermal fragments of mealworms from the rodent's original meal.

Attractiveness of faecal pellets to the dung beetles varied with the mammal species producing them. Appendix 3 shows that whereas rodent faecal pellets attracted a high diversity of dung beetle species, treeshrew pellets attracted only two (*On. lindaae* and *Oc. thai*). Analysing the number of beetle visits per hour further emphasised the relative unattractiveness of *T. belangeri* pellets to dung beetles, compared with the rodent pellets (Figure 8). The most attractive faecal pellets were those of *M. surifer* .

**FIGURE 8:**
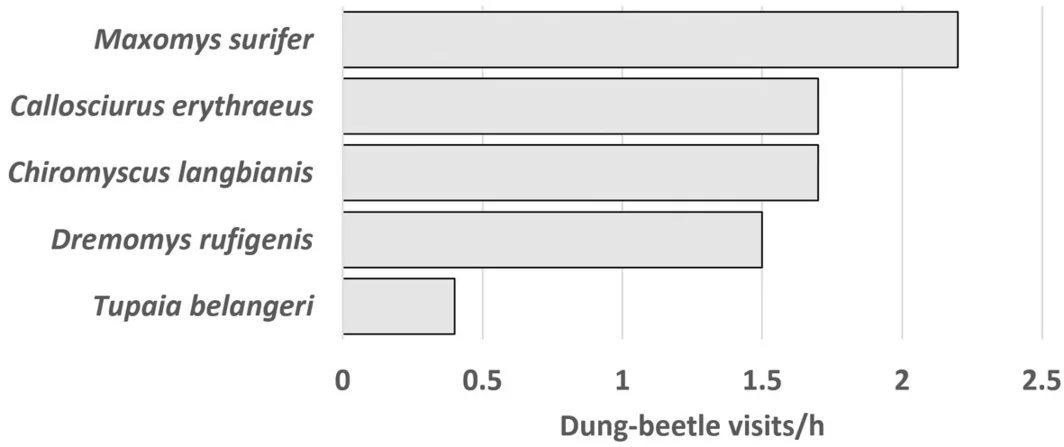
Visitation rates of dung beetles to faecal pellets of the primary seed‐dispersing mammal species, as a crude indication of relative attractiveness. Observation periods averaged 16 h per species.

This work resulted in the description of seven new species: 1 for northern Thailand (*Onthophagus sangwalus*) by Masumoto et al. (2008a), 5 for southern Thailand (*On*. *baenzigeri* (later found also in northern Thailand), *On. chumphonensis*, *On. phatoensis*, *On. ranongensis* and *On. thaitai*) by Masumoto et al. (2007, 2008a, 2008b) and 1 for N Borneo *On. koechlei* by Masumoto et al. (2011).

## 5 Discussion

### 5.1 Primary Seed Dispersal

Our findings demonstrate that several small‐mammal taxa—widespread and abundant throughout the ranges of *Tetrastigma* lianas and their *Rafflesiaceae* parasites—consumed *Rafflesiaceae* fruits and ingested seeds. Furthermore, the mammals were capable of dispersing significant quantities of intact seeds across the forest floor at appreciable densities in their faecal pellets.

Previously confirmed consumers of *Rafflesiaceae* fruit comprised the large treeshrew ( *Tupaia tana* ) and the plantain squirrel *(Callosciurus notatus)* (Emmons et al. 1991). Our study reaffirmed treeshrews and squirrels as consumers of Rafflesiaceae fruits and thus potential seed dispersers, with the capture of four specimens each of *T. belangeri* (Figure 5F) and *D. rufigenis* (Figure 5D) in traps over *S. himalayana* fruits.

Previously reported evidence for the involvement of Muridae species in Rafflesiaceae seed dispersal came from indirect observations of toothmarks on partially consumed fruits of *S. himalayana* (Elliott 1992) and *Rh. lowii* (Bӓnziger et al. 2007). Our study adds four Muridae species to the list of confirmed Rafflesiaceae fruit consumers and likely primary seed‐dispersers.

The duration of seed dispersal during endozoochory (i.e., the time between expulsion of the first and last seed) ranged from 20 h ( *T. belangeri* ) to 96 h ( *M. surifer* ) (from data in Table 3). Given these extended expulsion periods, potential dispersal distances far exceeded the known home‐range limits of the species involved. Consequently, we constrained the maximum displacement distance (the distance from the source Rafflesiaceae fruit to the point of seed expulsion), as the square root of each species' home‐range area. To account for the high variability in published home‐range sizes across different seasons and habitats, we utilised mid‐range values, synthesised from multiple sources. The estimated potential displacement distances, thus derived, ranged from 84 m for female *M. surifer* to 158 m for male *D. rufigenis* (Table 4).

**TABLE 4:** Estimates of *S. himalayana* seed displacement and encounters with *Tetrastigma* hosts across small‐mammal home ranges.

Species	Home range (ha)	Maximum displacement distance (m)^d^	Ground foraging time (h per 24‐h period)	Seed expulsion duration (h)^h^	No. of foraging periods	Estimated no. *Tetrastigma* stems in home range^i^
*Tupaia belangeri* ♂	1.5^a^	122	13^e^	19.8	1	23
*Tupaia belangeri* ♀	1.0^a^	100	13^e^	19.8	1	15
*Dremomys rufigenis* ♂	2.5^b^	158	4^f^	32.6	2	38
*Dremomys rufigenis* ♀	0.6^b^	77	4^f^	32.6	2	9
*Maxomys surifer* ♂	0.8^c^	89	11^e^	95.7	4	12
*Maxomys surifer* ♀	0.7^c^	84	11^e^	95.7	4	11
*Niviventer fulvescens* ♂♀	No data	No data	5^e^	73.3	3	Indeterminable
*Chiromyscus langbianis* ♂♀	No data	No data	< 1^g^	30.4	2	Indeterminable

^a^ Wang (2012).

^b^ Lurz et al. (2013) for closest analogous species *C. erythraeus* .

^c^ Walker and Rabinowitz (1992).

^d^ Square root of home range.

^e^ Suzuki et al. (2006).

^f^ Estimated from typical activity budgets of tropical sciurids and semi‐terrestrial foraging behaviour.

^g^ Almost exclusively arboreal.

^h^ From Table 3.

^i^ From Lü et al.'s (2009) estimate of 15 stems/ha.

Estimated daily ground‐foraging durations were derived from published activity budgets and adjusted to reflect the proportion of active time spent on the forest floor. These durations varied substantially due to species‐specific habits, ranging from about 1 h for the predominantly arboreal *C. langbianis* (Figure 5C) to 11 and 13 h for *M. surifer* and *T. belangeri* , respectively. Because the latter two species spend all or most of their active periods on the ground, they have far greater potential to ingest and disperse *Rafflesiaceae* seeds than the more arboreal species.

The duration of seed expulsion, following a meal of *S. himalayana* fruit, spanned from one to four activity periods (for *T. belangeri* and *M. surifer* , respectively). This suggests that *M. surifer* , for instance, could potentially deposit seed‐laden faecal pellets across its home range up to four times over, creating a widely dispersed seed rain across the entire area. Furthermore, among the species studied, only *M. surifer* nests and rests in ground burrows, whereas the others retreat to tree hollows to do the same. Consequently, *M. surifer* could continue to expel seeds in close proximity to *Tetrastigma* roots, even during periods of inactivity.

Adopting a conservative estimate of approximately 15 *Tetrastigma* stems per hectare (Lü et al. 2009), we estimated that between 9 and 28 host stems likely occur within effective seed‐deposition ranges of the species studied. These findings suggest that small mammals are capable of depositing Rafflesiaceae seed in close proximity to several potential new hosts, during typical foraging behaviour.

The relative effectiveness of each mammal species, as primary dispersers of Rafflesiaceae seeds, depends on a combination of several factors: (i) home‐range size (Table 4)—larger ranges are likely to include more *Tetrastigma* host individuals; (ii) time spent on the ground—some seeds expelled by arboreal species may remain on canopy structures away from *Tetrastigma* roots; (iii) gut‐passage duration (Table 3)—longer retention increases the likelihood of dispersal over greater distances and across multiple activity periods; (iv) number of faecal pellets produced per 24 h (Table 3)—higher output increases the extent and intensity of the seed shadow; and (v) the attractiveness of faecal pellets to dung beetles (Figure 8). A synthesis of these factors for each species is presented in Figure 9.

**FIGURE 9:**
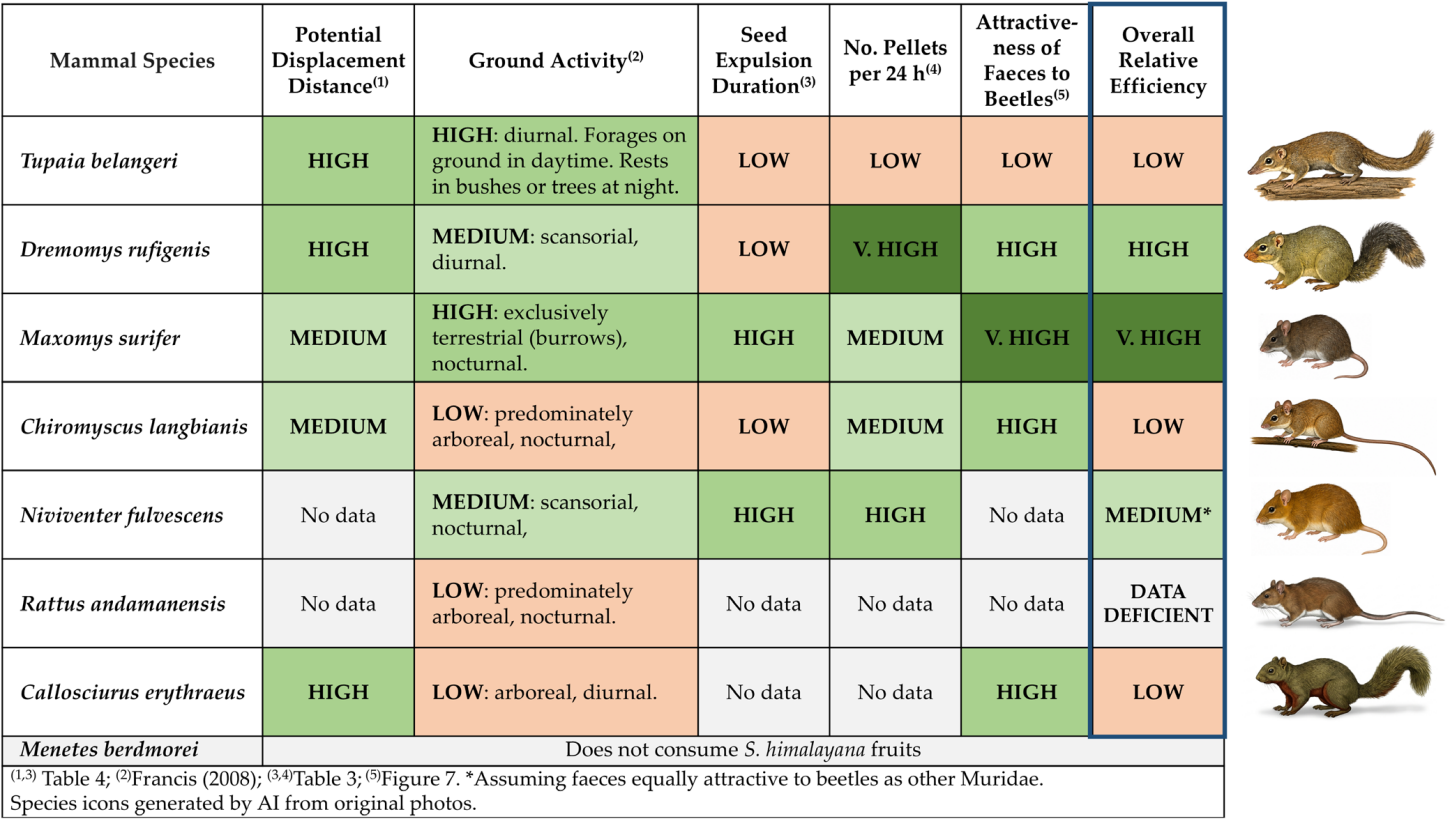
Synthesis of factors affecting efficiency of small mammal species as dispersers of Rafflesiaceae seeds. Classifiers (LOW–V.HIGH) are relative among the species.

From this synthesis, *M. surifer* emerges as potentially the most effective seed disperser. It forages exclusively on the ground at night, resting in burrows, up to 2 m deep, during the daytime (Wells et al. 2006). Thus, it could potentially deposit seed‐laden faeces very close to *Tetrastigma* roots. It expels seeds slowly, over up to four foraging periods (Table 3). Consequently, seeds are likely to be dispersed widely and evenly across an individuals' entire home range. The other 3 murids, in the present study, are likely to be less efficient nocturnal dispersers. *N. fulvescens* (Figure 5B) divides its foraging time between the understorey vegetation and the ground (Wilson et al. 2017), whereas *R. andamanensis* and *C. langbianis* are both predominantly arboreal (Francis 2008) (although capture of the latter on the ground in the present study indicates occasional ground visits).

During the day time, *D. rufigenis* (Figure 5D) assumes the role of primary seed disperser. It expels substantially greater numbers of seed‐laden faecal pellets than the rats and the treeshrew, over > 32 h following seed ingestion (Table 3). The species is considered to be scansorial, dividing its active hours between the ground and low understorey vegetation (Francis 2008). Although only about half of its foraging time is spent on the ground, seeds would be dispersed over two foraging periods (Table 4) across its relatively large home range (Table 4), thus increasing the probability of seed deposition near multiple potential *Tetrastigma* host lianas. Other squirrels, Pallas's squirrel ( *C. erythraeus* ) and the Indochinese ground squirrel ( *M. berdmorei* ) were not attracted into traps placed over *S. himalayana* fruits. In ad hoc, ex situ feeding trials, *C. erythraeus* consumed *S. himalayana* fruit, whereas *M. berdmorei* rejected it, indicating unpalatability. Furthermore, the predominantly arboreal niche of *C. erythraeus* (Nowak 1999) limits the species' potential as a primary disperser of ground‐dwelling Rafflesiaceae species. Consequently, primary dispersal of Rafflesiaceae seeds may be restricted to a few specialist Sciuridae taxa, rather than being a family‐typical capability.

*T. belangeri* is omnivorous but predominantly insectivorous (as evidenced by its dentition (Schwermann et al. 2023)). However, it was clearly attracted to and consumed *S. himalayana* fruits. The species occupies relatively large home ranges. It spends the daytime (around 13 h) foraging on the ground (Suzuki et al. 2006), retreating to tree hollows mostly at night to rest, nest and escape predators (Majumder and Agarwala 2015). Seeds would be expelled over a single foraging period after ingestion, in small numbers of comparably large faeces (Figure 5E). This would generate a more clumped seed shadow than that produced by the rodents. More importantly, dung beetles were only weakly attracted to the larger *T. belangeri* faeces (Figure 8). Consequently, although we consider *T. belangeri* capable of dispersing Rafflesiaceae seeds, it may not do so as efficiently as the rodents. However, other *Tupaia* species may be more effective, particularly *T. tana* , which Emmons et al. (1991) reported eats *Ra. keithii* fruits in Sabah. This species often nests in underground cavities at the base of large trees (Wells et al. 2006), potentially depositing seed‐laden faeces close to *Tetrastigma* root zones.

Although caution should be applied when interpreting this synthesis (since feeding trails were performed on a single animal of each species), the combined evidence suggests that *D. rufigenis* and *M. surifer* are the most plausible and prolific primary seed dispersers of Rafflesiaceae seeds, during the daytime and night time respectively, compared with the other species studied. Their geographic distributions and habitat preferences strongly coincide with those of many Rafflesiaceae species. However, both are absent from the Philippines, widely regarded as a centre of diversity and endemism for *Rafflesia*. Furthermore, the distribution of *D. rufigenis* does not extend south of central Peninsular Malaysia. Consequently, ecologically analogous species, with similar diets, foraging behaviour and ranging patterns, and wider distribution, should also be investigated as potential seed dispersers of Rafflesiaceae species.

Two other predominantly terrestrial murids, common in Rafflesiaceae sites, and with similar dietary and burrowing habits are *M. rajah* and *Berylmys bowersi* (Wells et al. 2006). Their distributions extend beyond Thailand into Malaysia and Indonesia, but they are also absent from the Philippines. In the Philippines, due to high island‐endemicity, murid involvement in seed dispersal is likely to comprise a diverse guild, including multiple *Rattus* and *Apomys* species. *Rattus everetti* (Mindanao) and *Bullimus luzonicus* (endemic to Luzon, which supports the most species‐diverse *Rafflesia* assemblage in the archipelago) deserve particular attention (Heaney et al. 2016).

Potential sciurid analogues for *D. rufigenis* include *Callosciurus notatus* , a known consumer of *Ra. keithii* fruit (Emmons et al. 1991), as well as the highly terrestrial *Lariscus insignis* and *Rhinosciurus laticaudatus* , in Peninsular Malaysia, Sumatra and Borneo; *Rheithrosciurus macrotis* and *Sundasciurus everetti* (formerly placed in *Dremomys*) in Borneo (lowlands and uplands, respectively) and *Sundasciurus philippinensis* (Mindanao) in the Philippines (ThoringtonJr. et al. 2012).

### 5.2 Alternative Primary Dispersers

We concur with Emmons et al. (1991) that large mammals would be suboptimal seed dispersers, because they would deposit seeds in relatively few, large dung piles rather than distributing them more evenly across the forest floor. In contrast, rodents and treeshrews produce numerous small faecal pellets, resulting in a more diffuse spatial pattern of seed deposition. Moreover, the larger, softer dung of large mammals differs markedly in consistency from the compact pellets of rodents and scandents. Consequently, it attracts a different assemblage of dung beetles.

Considering the suitability of ants as potential primary dispersers: despite Pelser et al.'s (2013, 2018) observations of ants interacting with seeds in a decaying fruit of *Ra. philippensis* and *Ra. speciosa* in the Philippines, we consider them unlikely to be effective dispersers. HB only once observed large numbers of ants on the decomposing pulp of a naturally split open *S. himalayana* fruit. However, seed transport by the ants was not observed. Emmons et al. (1991) noted the absence of ants and termites on the broken fruit of *R. keithii* in Sabah, Malaysia and wrote: ‘indeed its sticky pulp would probably repel ants’. Furthermore, seeds of myrmecochorous plants are typically 3–9 times larger than those of *S. himalayana* (Pfeiffer et al. 2010) and they usually possess elaiosomes (nutrient‐rich seed appendage, attractive to ants). Pelser et al. (2013) speculated that the chalazal swelling of *Rafflesia* seeds (proximal lobe Figure 2D) might constitute an elaiosome. However, Bouman and Meijer (1994), stated that such structures were not elaiosomes in their detailed morphological treatment of Rafflesiaceae seeds by SEM.

Furthermore, ants disperse seeds over very short distances (∼1 m; Gόmez and Espalader 1998), which would rarely be sufficient to infect many new *Tetrastigma* hosts. HB also observed that single ants frequently visited the faecal pellets of primary dispersers, antennating them for some time before leaving without carrying away the pellets.

To verify if ants play any significant role in Rafflesiaceae seed dispersal, a comprehensive examination of their behaviour in and around the fruits would be required, to determine (i) how they interact with the seeds (as dispersers or predators), (ii) over what distances they might transport them and (iii) where they might deposit them (within or outside nests). The possibility that ants may be capable of re‐infecting hosts, where no mammalian fruit consumers are active should also be investigated.

### 5.3 Secondary Seed Dispersal

Dung beetles may facilitate infection of a *Tetrastigma* host liana by Rafflesiaceae seeds in two ways: (i) burial of seed‐laden faeces of primary dispersers and (ii) seed release by disaggregating faecal pellets.

Our study revealed that dung beetles rapidly locate and bury the seed‐laden faecal pellets of the primary dispersers, rarely immediately, but mostly after rolling them over short distances. Dung‐beetle assemblages are structured by dung type, size, consistency and volatile composition (Nichols et al. 2009). The small firm rodent pellets attracted guilds (around 20 species in each location) of small, non‐balling rollers, which buried the pellets to observed depths of up to 7 cm, increasing the likelihood of placing them near *Tetrastigma* roots. The faeces of *T. belangeri* elicited a different response. Only two beetle species (*Oc. thai* and *On. lindaae*) were observed handling them on two occasions (Appendix 3). Such limited attention might stem from the faeces' large size and soft consistency (Figure 5E), which necessitated balling before rolling—rendering them a non‐preferred food source to typical non‐balling rollers. However, it is conceivable that such faeces might be exploited where preferred, smaller, firmer, rodent pellets are scarce. Both *Oc. thai* and *On. lindaae* were the most abundant beetle species at the northern Thailand site and are two of only three species common to both Thailand sites (Appendix 3). Although *T. belangeri* faeces received little attention from dung beetles during daytime observations, they were removed overnight (unobserved). Further nocturnal observations are therefore needed.

Dung beetles feed primarily on microbial biomass and the fine organic particles that constitute the bulk of rodent dung. The maximum particle size that can pass into a dung beetle's digestive tract is typically 2–130 μm diameter (Holter and Scholtz 2007), being constrained by the proventriculus (a foregut section that grinds food and regulates passage into the midgut). This precludes ingestion of Rafflesiaceae seeds (150–400 μm). Consequently, mechanical disaggregation of faecal pellets (rather than ingestion) is the mechanism by which dung beetles release Rafflesiaceae seeds into the environment. This was verified by our ex‐situ experiment using *On. avocetta* (the largest species in our study). The rapid disaggregation of faecal pellets and release of *S. himalayana* seeds would accelerate the process of seeds coming into intimate contact with *Tetrastigma* rootlets.

### 5.4 Infection

So far, attempts to induce germination of Rafflesiaceae seeds *ex situ* have failed (Mursidawati et al. 2014; Wicaksono et al. 2016). Both germination and infection of *Tetrastigma* hosts remain largely unknown, but probably involve (i) mechanical wounding of *Tetrastigma* fine rootlets to create entry points and (ii) chemical cues from microbial activity to stimulate germination and infection. These two processes are intimately linked since wounding could release the chemical germination cues from the host.

Our research raises the question of the role that seed dispersers might play in wounding *Tetrastigma* roots. In this respect, further detailed investigations of the burrows of *Maxomys* spp., are needed, since their strong teeth and claws are easily capable of wounding *Tetrastigma* roots. The possibility that dung beetles could wound very fine rootlets and root hairs with their strong toothed forelegs, as they drag faecal pellets down into the soil, also warrants further investigation. Bänziger (1991, 2004) also proposed that the soil fauna, mainly arthropods and nematodes, might gnaw, scratch and suck from *Tetrastigma* rootlets, causing microscopic wounds, where *Rafflesia* seedlings could penetrate.

Recent research has begun to reveal complex interactions between the microbiomes of *Rafflesiaceae* parasites and their hosts, and the metabolites they produce (Molina et al. 2021, 2024, 2025). However, this research has not yet identified any such metabolites capable of inducing Rafflesiaceae seed germination and host infection, despite the testing of numerous chemicals (Molina et al. 2021).

Molina et al. (2024) proposed that the seed microbiome comprises bacteria, acquired from the original fruit and its host. Our findings add complexity to this scenario, since the passage of seeds through the digestive tracts of small mammals, their subsequent burial in the microbe‐rich organic soil horizon and their eventual manipulation by beetles (which possess a rich microflora, both internally and externally and transmitted from larvae to adults) may all modify the seed microbiome. Therefore, we suggest that the microbiomes of the seed‐dispersing mammals and beetles as well as the soil organic horizon and their metabolites should all be included in the search for potential germination cues.

## 6 Conclusions

In this paper, we present evidence that supports diplochorous seed dispersal in the Rafflesiaceae, shedding light on the complex and poorly understood life cycle of these extraordinary parasitic plants. Our findings validate earlier speculation (Meijer 1958; Emmons et al. 1991) that small mammals serve as primary seed dispersers. Although the distances over which such mammals deposit seed‐laden faeces are not long (80–160 m), their home ranges are nevertheless sufficiently extensive to potentially encompass multiple *Tetrastigma* stems (Table 4). Due to their burrowing behaviour and prolonged duration of seed expulsion, the *Maxomys* murids emerge as highly capable of depositing seeds in the rooting zones of host liana species.

Furthermore, we provide new evidence that dung beetles act as secondary seed dispersers. By burying and disaggregating the faecal pellets of primary dispersers, these insects would bring Rafflesiaceae seeds into intimate contact with the fine rootlets and root hairs of potential host *Tetrastigma* spp—the most likely infection routes due to their high surface area and thin, delicate outer layers, conducive to penetration. Dung beetles in the genera *Ochicanthon* and *Onthophagus* are identified as the most likely candidates for effective secondary dispersal.

Finally, our research suggests new avenues for investigation into the potential involvement of microbiomes and their metabolites in the quest to discover chemical cues that may trigger natural germination of Rafflesiaceae seeds—the ‘holy grail’ of Rafflesiology.

## Author Contributions

**Hans Bänziger:** conceptualization (lead), data curation (equal), investigation (lead), methodology (lead), project administration (lead), resources (lead), visualization (equal), writing – original draft (equal), writing – review and editing (equal). **Kimio Masumoto:** data curation (equal), investigation (equal), writing – review and editing (supporting). **Chainupon Suwannakunpaisan:** data curation (equal), investigation (equal), writing – review and editing (supporting). **Stephen Elliott:** conceptualization (equal), data curation (lead), formal analysis (lead), validation (equal), visualization (equal), writing – original draft (equal), writing – review and editing (lead).

## Conflicts of Interest

The authors declare no conflicts of interest.

## Data Availability

Data are available from DRYAD. https://doi.org/10.5061/dryad.2v6wwq04t.

## Appendix 1 A Summary of Pollination Mechanisms of the Target Rafflesiaceae Species—As a Prerequisite for Rafflesiaceae Fruiting

**Table:**

Species of flower	Species of pollinator	Dipterous family	Pollination mechanism	Source and comments
		Sex involved		
*S. himalayana*	*Lucilia porphyrina*, rarely other blow fly species	Calliphoridae. Females; rarely males	By circumambulating the underside of a male disc, a pollen smear is acquired from one or more anthers, delivering it later in the same way to a female flower (where the wet stigmatic fascia can reliquefy the smear if it dried while inflight in dry air).	Bӓnziger (2004). Pollination of *S. himalayana* f. *albovinosa* in NE Thailand was by female *Chrysomya pinguis* (Bänziger et al. 2000), *S. poilanei* in SE Thailand by female and male *C. papuensis* (Bӓnziger 2004). Analyses of volatile attractants see Bӓnziger et al. (2022).
*S. ram*	*Sarcophaga albiceps*	Sarcophagidae	As described for *S. himalayana* . When any males present, they used the flower as a rendezvous site, without entering the flower.	Bӓnziger and Pape (2004). Analyses of volatile attractants see Bӓnziger et al. (2022).
	*Sarcophaga nathani*	Females only		
	*Sarcophaga notabilis*			
	*Sarcophaga krathonmai*			
	*Sarcophaga saprianovae*			
	*Sarcophaga yunnanensis*			
	*Sarcophaga coei*			
	*Sarcophaga multivillosa*			
	*Sarcophaga ostindicae*			
	*Sarcophaga pattoni*			
	*Sarcophaga seniorwhitei*			
*Ra. kerrii*	*Chrysomya chani*	Calliphoridae	A pollen smear is acquired by advancing below the disc and entering one of the channels leading to its single anther, delivering it later to a female flower by circumambulating below the disc with its wet stigmatic fascia (where the smear can be reliquefied if already dry).	Bӓnziger (1991, 2004). For other *Rafflesia* species, see Beaman et al. (1988), Wee et al. (2018). Analyses of volatile attractants see Patiño et al. (2000, 2002), Jürgens et al. (2013), Bӓnziger et al. (2022).
	*Chrysomya defixa*	Females only		
	*Chrysomya pinguis*			
	*Chrysomya rufifacies*			
	*Chrysomya villeneuvi*			
	*Lucilia porphyrina*			
*Rh. lowii*	*Hemipyrellia ligurriens*	Calliphoridae	Analogous to that of *S. himalayana*	Bӓnziger et al. (2007). Pollination of *Rh. infanticida* (as *Rh. zippelii*), and *Rh. deceptor*, see Bänziger (1996, 2001). Smells very differently from *Rafflesia* and *Sapria*, see Patiño et al. (2000, 2002), Bӓnziger et al. (2022).
	*Hypopygiopsis fumipennis*	Females only		
	*Hypopygiopsis violacea*			
	*Chrysomya defixa*			
	*Chrysomya pinguis*			
	*Chrysomya rufifacies*			
	*Chrysomya villeneuvi*			
	*Lucilia porphyrina*			

## Appendix 2 Morphological Traits of Rafflesiaceae Fruits Consumed by Mammals. Circumference C (cm); Length L (cm)

**Table:**

Species	Size	Shape	Surface structure	Colour
*S. himalayana*				
Fruit	C 21–32; L 3.1–5.0	Flattened barrel, very slightly convex walls, capped by disc, diameter 6–8.5	Walls with very evident horizontal and vertical cracks, capping disc with trapezoid cracks	Blackish
Seed	0.6–0.65 × 0.29–0.33 mm; estimated number per fruit, 20.000	‘J’‐shaped	Reticulation clearly finer‐meshed than in *Ra. kerrii*	
*S. ram*				
Fruit	C 20–21; L 2.8–3	Flattened barrel, very slightly convex walls, capped by disc, diameter 4–4.5	Walls with ± clear horizontal and vertical cracks, capping disc with 4–8 large and some smaller trapezoid cracks	Blackish
Seed	0.58–0.66 × 0.28–0.34 mm; estimated number per fruit, 6000	‘J’‐shaped	Reticulation slightly broader‐meshed than *S. himalayana* but less than in *Ra. kerrii*	
*Ra. kerrii*				
Fruit	C basal 40–51, upper 25.6–34; L 7.5–11.5	Truncated cone‐like with constriction below top, capped by disc, diameter 6.1–11.8	Cracks all over but less evident than in *Sapria*	Red brown
Seed	0.86–0.99 × 0.39–0.44 mm; estimated number per fruit, 100,000	‘J’‐shaped	Reticulation broader‐meshed than in *Sapria* and *Rhizanthes*	
*Rh. lowii*				
Fruit	C 23–25 cm; L 3–5 cm	Side walls slightly converging from base to capping disc	Walls with horizontal and vertical cracks, capping disc with trapezoid cracks	Blackish
Seed	0.70–0.78 × 0.33–0.40 mm; estimated number per fruit, 10,000	± ‘J’‐shaped	Reticulation slightly less broad than in *Ra. kerrii*	

*Sources*: Bӓnziger (2004), Bӓnziger et al. (2007).

## Appendix 3 Observed and/or Captured Beetle Species Attracted to the Faecal Pellets of Primary Seed Dispersers in Three Locations Near Four Rafflesiaceae Species

**Table:**

Location and species	Beetle size (length, mm)	No. specimens seen rolling/burying	No. specimens in pit traps	Activity months	Mammal origin of pellets^a^
**N. Thailand—*Sapria himalayana***					
*Cassolus ponchaii*	3.5–5	0	1	XI	Ch
*Ochicanthon thai*	3.5–5	63	21	VIII‐XII	Ch + Ce + D + M + T
*Onthophagus avocetta*	13–16	21	2	VII‐X	Ch + D + M + *N* + *R*
*Onthophagus baenzigeri*	4.5–5	1	1	VIII+XI	D + *R*
*Onthophagus doisuthepensis*	4	22	1	VI‐XII	Ch + Ce + D + *N*
*Onthophagus frugivorus*	7	1	1	X + XII	D + M
*Onthophagus grandivigilans*	5	1	0	IX	Ch
*Onthophagus lannamiibun*	4	7	18	VIII‐XII	Ch + Ca + D + M + *R*
*Onthophagus lindaae*	6–8	41	11	VI‐XII	Ch + Ca + D + M + *R* + T
*Onthophagus maleengnaafon*	2–3.3	0	1	XI	M
*Onthophagus naroon*	3.2–4	4	31	X‐XII	Ch + Ce + D + M
*Onthophagus pseudoarai*	4	2	0	VIII	R
*Onthophagus sangwalus*	4.5–6	2	19	IX‐XII	D + M
*Onthophagus taayai*	5–6.5	8	4	IX‐XII	Ch + D + M
*Onthophagus thanwaakhomus*	4–5	5	2	X‐XII	Ch + D + M + *N*
*Onthophagus tongbantumi*	5	1	0	VIII	Ce
*Onthophagus trituber*	6	2	0	VIII	Ce
*Onthophagus* sp. near *punneeae*	6	1	0	VIII	Ce
*Panelus tonkinensis*	2–3	16	2	VIII, X, XII	Ce + M
*Parascatonomus maesaensis*	4–5	12	6	VI‐XII	Ce + D + M
*Sisyphus maniti*	3–6	10	67	IX‐XII	Ch + Ce + D + M
**S. Thailand—*Rafflesia kerrii* & *Sapria ram*** ^**b**^					
*Copris agnus*	15–20	0	3	II	M
*Copris spinator*	13–17	0	3	II	M
*Ochicanthon thai*	3.5–5	1	2	VIII	R
*Ochicanthon* sp. 1	—	0	1	VIII	R
*Onthophagus baenzigeri*	4.6–5	6	0	III	Ce
*Onthophagus chrysurus*	6.5–9	0	6	VIII	R
*Onthophagus chumphonensis*	4	0	5	II	M
*Onthophagus deflexicollis*	4.5–5.5	1	0	III	Porcupine
*Onthophagus lindaae*	6–8	12	1	II, III	Porcupine, M
*Onthophagus mentaveiensis*	7–7.5	0	2	VIII	R
*Onthophagus nampatensis*	11–12	1	1	VIII	R
*Onthophagus obscurior*	7–8.5	1	0	III	Porcupine
*Onthophagus orientalis*	9	1	0	IX	Ce
*Onthophagus phatoensis*	9–11	0	3	II	M
*Onthophagus ranongensis*	3.5–5	11	28	VIII, IX	Ce + *R*
*Onthophagus* sp. 4	—	0	1	II	Ch
*Onthophagus* sp. 8	—	0	1	VIII	R
*Onthophagus* sp. 13	—	2	0	III	Porcupine
*Parascatonomus thaitai*	5	0	1	VIII	R
*Sisyphus thoracicus*	6–8	11	9	II, III	M, porcupine
**E. Malaysia (N. Borneo) *‐ Rhizanthes lowii***					
*Ochicanthon maryatiae*	7	1	No trapping	I	*Callosciurus* sp.
*Ochicanthon kikutai*	7	1	No trapping	I	*Callosciurus* sp.
*Onthophagus koechlei*	3.5–5	9	No trapping	I	*Callosciurus* sp.

^a^ Ch, *Chiromyscus langbianis*; Ce, *Callosciurus erythraeus*; D, *Dremomys rufigenis*; M, *Maxomys surifer* ; N, *Niviventer fulvescens*; R, *Rattus andamanensis* ; T, *Tupaia belangeri* .

^b^ One host infected by two Rafflesiaceae genera.
